# The Circulation of the Blood

**Published:** 1854-07

**Authors:** 


The talented author of the following excellent article administered to us a fatherly reproof, in the pages of the Boston Journal some time since, for using the weapons of ridicule, and satire, in a notice of Mrs. Willard's theory. We accepted the lecture with a sincere resolution to sin no more.

But it seems that even Dr. Chandler must sometimes laugh, and we have no doubt that our readers, after perusing the following pungent and pithy article, will (so far as Dr. C's reproof to us for levity is concerned) call to mind the old adage of a certain dispute as to complexion, between a pot and a kettle. H.

*The Circulation of the Blood.* (Communicated for the Boston Medical and Surgical Journal.)

The doctrine of Mrs. Willard, whether true or false, when first announced was comprised in a theorem of bold and definite outlines; and, as if abjuring all ambiguity, the declaration that "the chief motive power of the blood is in the lungs," which might have sufficed, was surmounted by another—"and not in the heart." So far, all is well, and well becomes the champion of a newly-discovered truth. But truths are more or less recondite; some requiring only to be presented to the mind of ordinary intelligence, to insure belief; others demanding elaborate detail and reiterated illustration, to be made intelligible. To the latter class, the truth, of which Mrs. Willard claims to be the discoverer, evidently belongs.

The questions involved in this theory are of grave import; and I dissent from the opinion of one of the correspondents of the Journal, that, true or false, they can be of no use to practical medicine. Mrs. Willard is justified in her assertion, assuming her doctrine to be true, that "it may be made available, in innumerable cases, both for the preservation of health and the cure of disease." Unfortunately, however, for the cause of truth, there has been no small amount of temptation to meet the announcement of her discovery with badinage, rather than with argument. The pages of the Journal, in her own communications, no less than in those from Dr. Cartwright, furnish abundant material to illustrate what I mean. I might specify many examples; for instance, in a late number, she speaks of "the truth of the God of nature, for a season committed to her weak hands." A sore trial, I fear, to Dr. Hunt's continence; and I trust the publisher erased the passage from the doctor's copy. The well-trained and well-stored mind of the fair theorist would supply the appropriate comment at once, could she separate herself from the supposed discovery, and its anticipated halo. In regard to Dr. Cartwright, she might exclaim, "save me from my friends, and leave my enemies to me." I need only refer to former numbers of the Journal, for examples of indiscreet and premature laudation, so ineffably absurd and ridiculous, that we may well marvel whether, from Dr. Cartwright, it could have been anything less than irony.

Had Mrs. Willard published her discovery in any other terms than those implying veritable demonstration; had she clothed it in the graceful garb of suggestion or inquiry—an appropriate costume, surely, for a lady on her first appearance in a department of science, which must have shared quite sparingly in the multifarious labors of her active life—she would have met with no repulse. Her own premature appropriation of the glories of her supposed discovery; her unwarranted assumption, that the methods of resuscitation, and the art of preserving and restoring health by the agencies of exercise and pure air, now and long since understood and practiced by every tyro in physic, were the results of her own discovery; her audacity in claiming all these as her own timely gratuity to the world, just on the eve of suffocation—surely, these are no light provocatives; and may at least palliate the discourtesies of some of the correspondents of the Journal. In my own professional career, I have served, as doubtless thousands have done before me, in the humble capacity of prompter to some scores of urchins, at their "entrance on the stage," where their appropriate business was to "play their parts," but who were doggedly bent on forgetting them. Having no tact at gentle persuasives, and no *meal* in my *mouth*, I have "blown them up" at once; and candor compels me to confess, the malaperts have seldom failed to "give me as good as I sent."

Such, I apprehend, are among the causes which have prevented Mrs. Willard's theory from receiving the notice, which it may possibly deserve from competent physiologists. But what is her theory? It is noticeable that the phraseology of her "epitome," contained in a late number of the Journal, is somewhat less clear and definite than her original theorem, which was that "the chief motive power of the blood is in the lungs, and not in the heart." In the "epitome," it is thus modified—"the chief motive power of the blood originates in the lungs, in consequence of respiration." Is the import of the first, identical with the last? And does not her omission of the latter clause in her original theorem, "and not in the heart," and her use of the phrase, "originates in the lungs," as she has it in her "epitome," savor of some embarrassment in adapting it to all the exigencies of the circulation? If the epitome was intended to elucidate, and furnish an intelligible rationale of her doctrine, it is clearly a failure. It still remains a naked theorem; and a bare assertion that "the chief motive power of the blood is in the lungs and not in the heart." The "explanation," contained in the epitome, "which she had some time felt to be necessary," furnishes merely the assertion that "a portion of the water in the blood is there changed to vapor, and the volume of the blood becomes so expanded that it must move. At the capillaries, and in the veins, it is condensed by the effect of the external air." This is the sum of her rationale of the circulation; the consummating elucidation of "the truth of the God of nature, committed for a season to her weak hands." Having thus finished her labor; having given to physiology a new domain; having taken formal possession, not in Ferdinand's, but in her own, name, I trust no Americus Vespucius will rob it of its appropriate designation, *Willardia.* Columbus, after exclusive devotion of himself, for a long series of years, to the demonstration of his hypothesis, that a counterbalancing continent would be found in the west, indicated, as he thought, not only by general analogies, but by incidental evidence, "explained" his theory at last, by planting the feet of his incredulous followers on terra firma. "The mission which God has now crowned with success, was committed to *my hands* by Himself," was an exultation not only tolerated, but responded to by every intelligent and liberal mind. Did he indulge in this language, while inspecting the strange fragment of drift-wood found on the shores of the East?

In justice to Mrs. Willard, however, ought we not to admit that she may have been the first to notice a fragment of *drift-wood*, hitherto unobserved, on the field of physiology, and possibly of no small import in physiological research? I recollect nothing in the history of theorizing, on respiration and the circulation, which militates against her claim to originality in the suggestion, that the process of oxygenation and decarbonization of the blood, in the lungs, by the extrication of caloric, and the consequent expansion of its volume in the capillary vessels, may have an agency in the movement of the blood from the lungs, toward the left heart. If so, in all other regions, where aortic terminations and venous radicals are to be found, affording any facilities for the access of caloric, especially in the skin, this expansion may possibly contribute something, as "available" force, to capilary and venous circulation. This suggestion, or discovery, if it be substantiated, which is not impossible, is a creditable achievement, in which Mrs. Willard may justly feel complacency; and for which no *man*, "woman" though she be, may reproach her. The correspondents of the Journal have merely objected to the transformation of her islet, by her own mental mirage, into a mighty continent; to her hypothetical metastasis of the chief motive power; to her transfer of empire from Rome to Constantinople; and, especially, to her edict, that on her own theory, as on a pivot, the entire interests of humanity shall hereafter revolve!

It is certainly unfortunate that the clearness and definiteness of Mrs. Willard's theory have been much obscured, in her progressive postulations and "definitions of her position." Does she mean, by "motive power," veritable propulsive force; the *proximate* agency, by which momentum was imparted to "the stone from the brook," that smote the Philistine? This would be in accordance with her original theorem; "the chief motive power of the blood is in the lungs, and not in the heart." Or does she mean the *remote* causes, to be found in David's patriotism and allegiance to his Maker, prompting "in the same direction"? The latter has some adaptation to the theorem of the "epitome," which is worded—"the chief motive power of the blood originates in the lungs, in consequence of respiration." Although this variation in the form of her theorem contains no recantation, it admits of equivocation. Respect, however, for Mrs. Willard's consistency, as a philosopher, compels us to regard it as identical with the original.

Before considering the adaptation of Mrs. Willard's theory to the known principles of natural science, we should briefly advert to the settled opinions of physiologists, on the circulation of the blood. The heart, then, is regarded as the efficient agent, which propels its contents, by the contraction of the muscular walls constituting its ventricles; from the left, through the aorta and its branches, to the whole body; from the right, through the pulmonary arteries, to the lungs. The structure, size, firmness, position and relations of this organ, seem to fit it perfectly for the office assigned to it; and we look in vain for any other point in the circulation, which may furnish the indispensable facilities required, as a seat of any adequate amount of motive power. All the predecessors of Mrs. Willard, since the days of Harvey, have agreed in this, that the contraction of the ventricles of the heart is the chief propulsive power of the blood. Many have supposed that the *diastole*, no less than the systole of the heart, has an important agency in the circulation. The plenitude of rich material, and the highly wrought structure of the heart, must furnish it with the elements of great power; and it is demonstrable that its relations are so adjusted that it may act in a direction to *dilate* the ventricles. The fact having been established, that the systole and diastole of the living heart may continue, for a limited time, independently of the influx of any fluid, the inference is unavoidable that the diastole is not a *passive* result. Consequently, just in proportion to the power of the muscular action of the heart, effecting dilatation of its cavities, where a vacuum can only be averted by the influx of blood, will be the atmospheric pressure; operating on the vascular system by propelling its contents, "directed by the vascular system," into the dilating cavities; the heart, by a double function, thus propelling the blood through the arteries, by its systole, and imbibing it from the veins by its diastole.

Generally, the arteries have been regarded as exerting a small degree of propulsive force, both by the properties of contractility and elasticity appertaining to their coats. There can be no doubt of the fact, that these properties have an agency in adapting the caliber of the arteries to the varying quantities of blood, expelled by each systole of the heart; thus indirectly, at least, co-operating with the force of the heart. Physiologists, however, have been divided in their opinions, on the subject of capillary and venous circulation; some regarding both these portions of the vascular system as capable of propelling their contents, independently of the heart's action; others making them equally passive with the arteries; while others, I think with good reason, claim, both for capillaries and veins, the power of co-operation with the heart, in circulating their own contents. The fact, well authenticated, that a fluid injected into the aorta of the dead body, by moderate force, may be driven through the double capillary systum of the intestines and liver, and be made to flow out of the veins in full stream, is sufficiently conclusive to show, that, in the living subject, the action of the heart might suffice to propel the blood through the whole circuit of arteries, capillaries and veins.

Much speculation has been indulged, in regard to certain attractive forces, or affinities, supposed to co-operate in effecting circulation; such as the mutual affinities between the carbon in the contents of the pulmonary arteries, and the oxygen in the air-cells; and in the other extremity, the attraction of the tissues to be repaired, for the nutritive elements contained in the aortic terminations. Nevertheless, the doctrines of physiologists on the subject of the circulation, as commonly received and understood, claiming that the chief motive power of the blood is in the heart, are in harmony with the laws of natural science, and admit of an intelligible rationale.

The action of the heart being thus claimed by her predecessors, as the chief motive power of the blood, effecting circulation by a process which has been clearly explained, and is well understood, Mrs. Willard claims that "the chief motive motive power of the blood is in the lungs, and not in the heart"; and in her "epitome," has added, by way of "explanation," that "a portion of the water in the blood is there changed to vapor, and the volume of the blood becomes so expanded that it must move." If the motive power of the blood be in the lungs, it must have some point, or basis of propulsive action; and this basis must be the pulmonary tissue. The effective force is claimed by Mrs. Willard, to be expansion of the volume of the blood, by caloric; the capillaries of the lungs constituting, of necessity, the boilers where this expansion is effected. Being no "mechanician," but a "mere mechanic," I feel some timidity in suggesting the inquiry, whether "action and reaction" may not be as "equal" in physiology as in mechanics? If so, the pulmonary tissue being the seat of the expansive force, which is to propel a mass of blood, weighing probably more than thirty pounds, and the portion of the fluid, to be expanded in the lungs, being contained in vessels of small calibre, with coats of extreme tenuity; and these vessels being imbedded in a congeries f mere air-cells, also of extreme tenuity; the thirty pounds of blood requiing to be urged onward with an impetus that may overcome formidable obstacles in every direction; what will be the effect of this expansive force on the vascular and cellular tissues of the lungs? Do these tissues afford an adequate base, for sustaining the reaction of all this propulsive power; It may be fancy—but my own perceptions are teeming with frightful visions of bursting boilers, and gushing crimson.

What is the rationale of Mrs. Willard's process? Notwithstanding the "epitome," she leaves us to *contrive* the answer. Is it moved from the pulmonary capillaries, to the opposite system of aortic terminations, by the process of evaporation; "where it is condensed by the effect of the external air," and returned to its starting point, like the dribbling of the pot-lid, into the boiling vortex below? Or do the successive portions of blood, heated in the capillaries of the lungs, make their way in serial order through the denser mass which occupies the heart and large vessels? But here, a formidable obstacle, in the law of gravitation, is interposed, in the fact that the position of the different portions of the blood, in relation to each other, would render such interchange of occupancy impossible. Will a fluid *rise* through a medium less dense than itself; or *fall* through one more dense? Admit the possibility that position might interpose no obstacle to its progress, to the cavities of the left heart; to the arch of the aorta; would it plunge through the descending aorta, and the femoral arteries, to the pedal extremities? If so, it must be in violation of the laws of gravitation, to which the blood of the living body, in the relations of its different positions to each other, is no less amenable than the turbid waters of the Mississippi. Does "caloric," by rarefying the capillary portion, propel the aggregated columns of blood, contained in the entire vascular system, onward in its circuit, like the agency of exploding powder in the breech of the musket? Then, manifestly, to say nothing of the inappropriate material constituting the barrel, nor of the complex disproportions of its calibre—the breech should not be made of sponge-cake! If the blood had neither weight nor dimension, like hypothetical nervous fluid, or were resolvable into a medley of partialities and antipathies, possibly we might construct a "piece of apparatus," with complications of positive and negative poles, that would elucidate the misty rationale of Mrs. Willard's theory. Unfortunately, the blood has palpable materialism, and abounds in downright heaviness. Dead or alive, it has no active mood, but is utterly passive. It will not move, though it may be moved. The motive power must appertain to the lever, and not to the weight; to the pack-horse, and not to the burden.

The *burden* of the ponderous flood, which fills and sustains the solid organism of the living body; which scales acclivities with impetuous rush, and bursts its inclosures, if an impediment interposes in its path, is no transcendental sublimation, to be wafted on the wing of a butterfly, or to be urged onward, in solid columns, by motive impotence. For what other purpose was the solid brawn of the heart provided in such munificence; and wherefore its transcendently elaborate organization? "That its beat might be made available force, in the same direction"—that it might add its little quota of impulse to the *vis a tergo* of a musquito's blow-pipe! Shade of Harvey!!

If Mrs. Willard's theory is founded on truth, a "piece of apparatus" might be furnished, for its illustration, without troubling the "Smithsonian

Institution." It might consist of a human body, no matter how long dead, if organization remained entire. The vascular system, being emptied of its blood, and filled with tepid water, the air-cells of the lungs should be injected, through the trachea, with water of a higher temperature; the injection alternating with a suction pump, for its removal, in imitation of the respiration; thus heatingand expanding at intervals, the contents of the pulmonary capillaries, The contents of the blood-vessels should now be found coursing bravely, at every point of their circuit. Dr. Cartwright might propose to mend the experiment with a modicum of his "life;" but I counsel his fair protège not thus to compromise the claims of "caloric," in the anticipated triumph. The doctor has become slippery, occasioned, doubtless, by the lubrication consequent on his frequent handling of alligators, in his play between "life" extinct and "life" suspended. Don't trust him; for, truth to tell, his "life" of the blood is a less reliable motive power than her own "caloric." I am as unsuccessful as herself, in conceiving how his "life" can explain or make intelligible his rationale of the circulation.

What is life? The principle of life, regarded as belonging to the aggregate of animal matter, constituting a human body, can only be known to be present by certain phenomena appertaining to the body, during its union with this principle; and hitherto all attempts at description or definition of its essence, have signally failed. The manifestation of the union of this "life" with the tissues of the body, may be nearly comprised in the properties of irritability and contractility; but the rationale of this union, the ultimate process by which it is maintained, or dissevered, I fear is beyond the ken of mortal eye, beyond the scope of human investigation. Thousands of instances occur where the cause of death or the extinction of life, has eluded all the research that science and skill combined could command. The finger of God is applied, or withdrawn, and life ceases. This is the sum of our knowledge of the principle of life; and we know a little of its essance, as of the Divine existence.

But, admitting "life" to be the motive power, in virtue of its control over all the functions of the body, by which the relations it sustains to other objects may be changed; or by which the relations of its different parts with each other may be varied; still the doctor rejects the vitality of all other tissues, and claims the "life" of the blood itself, exclusively as the force by which it circulates. The body moves by the agencies contained within itself; locomotion is effected, and we are justified in supposing we understand the rationale of its performance. But the doctor's theory isolates the blood from all motive agencies, except its "life." Is the union of the "life" with the blood by chemical affinity? Or is it a solution; or a mixture? Is the "life" a biped or a quadruped? Is it a piston, a tractor, or a lever? Does it push, pull or pry? The doctor will do well to make terms at once, by resolving his own "life" into the "caloric" of his fair adversary; concluding with another distinguished theorist, that "heat is life."

There have been other projectors, among whom, if my learning be not at fault, was one Archimedes; who proposed to move the entire earth with "a piece of apparatus." Unfortunately he just lacked a fulcrum. Modern theorists are less fastidious. If the fulcrum is at hand very well; it not, they quietly finish their demonstration without it.

I beg the indulgence of the readers of the Journal, while I present a case of suspended animation, from my own practice, in which recovery was effected without artificial inflation of the lungs; and where the repudiated heart was the last organ to make manifestations of life, and the first to indicate its return. I do this not for the purpose of questioning the propriety of inflation as a means of resuscitation; nor intending to deny that it may be indispensable to success; nor that I would now employ the means, then used exclusively, though successfully otherwise than as auxiliary.

More than thirty years since, after a protracted attendance on a case of typhus fever, I closed my patient's eyes with my own hands, supposing him to be dead. An hour was spent in the needful arrangements for such occasions, when a friend of the patient took my arm for a walk, it being on a pleasant afternoon in September. We were soon re-called, in consequence of the alarm of the attendants, who observed, while engaged in the usual offices for the dead, slight contortion of the muscles of the face. On my return, however, the only indication of life I could detect, was the slightest possible tremor, in the region of the heart. No attempts were made to inflate the lungs, which were as quiescent as those of the fœtus in utero. A large kettle of water, placed over the fire, *after* the supposed death of the patient, had become heated, to boiling; its presence perhaps suggesting the treatment pursued; which was all comprised in enveloping the entire body, at once in thickfolded sheets, saturated with the contents of the boiling kettle, hot as the hands of the assistants could bear. The only motion given to the body, meanwhile, was lifting it on the hands, for the purpose of placing the folded sheets under it. This might have been repeated twice, or thrice; occupying, in the whole, at least thirty minutes, and probably more; making more than ninety minutes since the suspension of respiration. After this lapse of time, the tremor of the heart, previously so slight that I feared it might prove a mere illusion, became more evident; soon amounting to a distinct impulse, while the lungs were yet quiescent; but soon followed by slight convulsive struggle, speedily ending in re-establishment of respiration and circulation. To my great joy the patient recovered, and probably yet lives.

If we claim the *skin* as the seat of the "motive power of the blood," and that circulatlon is maintained by the combination of caloric with that portion of blood contained in its minute vessels, thus expanding its volume; what better confirmation of the theory can be furnished than the case above cited? The skin has an obvious advantage over the lungs, in firmness of tissue, if not in extent of surface, affording a more reliable basis for sustaining propulsive force. In sober truth, the skin does unquestionably, co-operate with the lungs, in some measure, in the process of oygenation and decarbonation of the blood; carbonic acid, if I mistake not, being one of the products of cutaneous exhalation. May not this and the following case, at least furnish a lesson on the folly of jumping at once at the conclusion, that the world is mainly indebted to Mrs. Willard's theory, illustrated by Dr. Cartwright's dissection of alligators, for the means of preserving health and restoring suspendid life?

I beg indulgence for the egotism of the following case, as it is evidently inseparable from the *subject*. Fifty years since, on a hot summer day, with a group of school-boys, during recess at noon, I made my way from the village school-house to the banks of a considerable river, a mile distant. There were from twelve to fifteen boys, some of them good swimmers. My own purpose was to become a pupil in the art. Although the water was deep, the gradual slope of the banks and the sluggish current making *wading* practicable, I improved it, as a preliminary trial; pushing onward, as I thought, cautiously, intending to turn when the surface reached my chin, and attempt swimming to the shore. Unfortunately, an abrupt inequality in the bed of the river took me by surprise, and suddenly engulfed me.

My own recollections of that incident do not correspond with those recently published, I think by some medical writer, in some journal or newspaper that has fallen in my way. In that case, occurring under like circumstances, the remarkable activity and rapid process of the memory were such, that all the forgotten incidents, actions and events of a life, were re-called to the mind, with entire distinctness; occupying, of necessity, but a few moments of time in the process. In my own case, there was only a confused apprehension of my condition; flitting visions of the tumult and alarm of my associates; a feeling of hopeless isolation from all human aid; an undefinable sense of constraint and discomfort; perhaps some bodily pain, but certainly no agony, though I was conscious I must die. In my own recollection, the time seems short, after my immersion, till I lost my consciousness. My next recollections are of bodily distress, surpassing in intensity, I think, anything I have suffered before or since. I found myself on the sand of the shore, in the hands of my companions, who were rolling me over its burning surface. It was a northern shore, and had a southern declivity, and was intensely heated by an unclouded meridian sun. My sensations cannot well be defined, but it was as if my body was sustaining the weight of a world, and every rotation it made seemed a redoubling of the intolerable agony of the pressure. At first, I was unable to intimate my wish to my deliverers, that they should desist. In due time, however, I succeeded in uttering a moan, to which they responded in joyful shouts, and redoubling their diligence in tumbling me over the sand. My restoration to life was soon completed. For what transpired during the interval of my unconsciousness, I can only depend on my recollection of the account rendered at the time, by that company of boys, among whom there were none more than 12 years old.

My associates were, soon after my disappearance in the water, aware of my peril, but for a short time surprise and terror disqualified them for action. They soon rallied, however, with the feeling that *a life* must be lost, unless rescued by themselves; and with the deliberation of men, formed their plan. To provide against the loss of another life in saving mine, they formed themselves into a line, those who could not swim taking the shore end, where a sapling afforded a sure support. In this manner, holding each others' hands, the line was extended from the sapling toward the place where I was found, on the bed of the river, my body recovered, and brought on shore. They found me apparently dead; certainly exhibiting to *them* no signs of life, the skin being cold and livid, with entire suspension of respiration. The description of my appearance, subsequently given by my associates to my father, who was a physician, though not in these terms, was in language of the same import, satisfying him that I had been thoroughly drowned. Some time was lost in devising further measures for my relief; the distance to the village, with an intervening strip of forest, precluding all hope of seasonable aid in that direction. My deliverers were earnest in commending to each other this and that expedient, till a master-spirit, in that band of miniature men, gained the attention of his associates, by quoting from his own little store of learning, an instance of recovery from drowning, effected by rolling the body in a barrell. But the barrel was unattainable. They decided promptly that the rotation of the body, and not of the barrel, was the gist of the matter, and proceeded at once to a practical illustration. The result having been given above, I have only to add, I am indebted for my life to the resources of a boy less than 12 years old, in planning and executing, with the aid of prompt and able coadjutors, an enterprise which, in France, would have been a better passport to "immortality" than to have been the author of an unproved "theory." My benefactor's name was James Jones, and he probably still lives in some of the Western States.

I beg the indulgence of the editors and their readers for thus occupying the Journal with these somewhat irrelevant details. And yet, may not space sometimes be afforded for the record of a noble deed; and of one, especially, which, though it might have done honor to mature manhood, was nevertheless prompted by the humanity, and achieved by the unaided resources of a band of tiny school-boys.

But, not wholly to lose sight of the purpose of these details; in my own case, the body, being taken from the water, cold, livid, motionless and insensible, does the history of such accidents justify the conclusion, that resuscitation would have been effected had my body been rolled on the *shaded turf* instead of the *hot surface of sand*, with its declivity toward an unclouded vertical summer sun? Admit that in the act of rolling the body, the chest might have been slightly contracted and expanded, could it have been sufficient to have made inflation a primary or "chief" item in the process of restoration? If caloric is the motive agency, operating by expanding the volume of blood in the capillaries, surely, in both cases above cited, the vessels of the skin were favored with access to motive material, furnished by the hot water and the heated sand, in sufficient abundance to effect the "expansion" of their contents, which Mrs. Willard claims to be the propulsive force of circulation. In the former case it would be madness to claim that inflation of the lungs could have preceded the phenomena which denoted returning life. Would she have the temerity to claim that the heat was transmitted through the walls of the chest, from the hot water, to the blood in the pulmonary vessels, thus effecting circulation? Inasmuch as the cutaneous vessels were manifestly provided, both by extent of surface and proximity to the hot water, with vastly better facilities for the expansion of their contents, than the vessels of the lungs, how will she prove that the skin in that instance, during the first moments of returning action, was not the seat of "chief" motive power? Has the organization of the minute vessels of the lungs a demonstrable peculiarity, by which it fits them exclusively for the expansion of their contents by caloric? Cannot heat be communicated to venous blood, independently of oxygen; and will it not expand it? Admitting expansion of blood in the capillaries to be the motive power, its seat could not be claimed exclusively for the lungs, It should be borne in mind, that there are other processes in the animal economy besides aeration of the blood, during which caloric must be evolved. Does not the nutritive material contained in the aortic extremities, in the consummating act of nutrition, as it escapes from the capillary, and effects coherence with its appropriate tissue, change from the fluid to the solid form; and can this change occur without evolving caloric? And why may not this constitute an equal claim, on the part of the aortic terminations, to the seat of motive power; the two poles of the circulation thus dividing the empire.

But what proof has Mrs. Willard, that the caloric evolved in the lungs is mainly expended in the rarefaction of the blood? May not pulmonary exhalation require an amouut of caloric for its purposes too large to leave anything more than the small remainder, which might be measured by the small difference between the temperature of arterial and venous blood, taken from any region of the circulation, near the aortic extremities? How much caloric does it require to evaporate an ounce of water, having already the temperature of the blood? I dare not trouble the "Smithsonian Institution" for the chemist to tell me; but I have just been reversing the process, by puffing my own blow-pipe against a window pane, in an unwarmed room, the thermometer considerably below zero—and the result is refreshing—a sheet of ice that would convert a glass of luke-warm water into a delicious beverage.

Have the relations of the nervous system to the circulation been sufficiently regarded, in the speculations of physiologists or pathologists, in their rationale either of healthy or diseased action? There are many indomitable mysteries in these departments, that must, probably, find their solution in the future discovery of laws, appertaining to the nervous system, hitherto hidden from scientific research.

In relation to the subject of resuscitation from apparent death, there is one fact which not only militates strongly against Mrs. Willard's assumption, that it can only be effected by inflation of the lungs, but puts at naught a goodly portion of the current theorizing on the proximate agencies, ultimate processes, or rationale, appertaining to many of the phenomena of healt,h disease, and even death! Many well-authenticated cases of resuscitation from apparent death have occurred, under circumstances that not only preclude the possibility that inflation of the lungs could have been accomplished, otherwise than as an effect of returning life; but all other known or supposed means of exciting any of the actions appertaining to life, have been equally inaccessible. The supposed victim of death has been wrapt in his shroud, his face covered, and the apparatus leading to the lungs compressed with hands; the body coffined and laid in state, where no careless footstep might cause a vibration of his narrow house, where no spoken word might break the stillness of the air, where no genial warmth might intrude. Yet here, after a long repose, in the very stillness of death, life, not only unaided, but sorely encumbered by human appliances, has risen from its quiescence, and resumed its sway!

J. L. CHANDLER.

St. Albans, Vt., February, 1854.

